# The Geographical Distribution of Health and Disease, in Connexion Chiefly with Natural Phenomena

**Published:** 1856-07

**Authors:** Alex. Keith Johnston

**Affiliations:** Corresponding Member of the Epidemiological Society of London.


### TRANSACTIONS

OF THE

### EPIDEMIOLOGICAL SOCIETY OF LONDON.

### Papers and Communications.

### ON THE GEOGRAPHICAL DISTRIBUTION OF HEALTH AND DISEASE, IN CONNEXION CHIEFLY WITH NATURAL PHENOMENA.

By ALEX. KEITH JOHNSTON, F.R.S.E., etc., Corresponding Member of the Epidemiological Society of London.

[*Read before the Epidemiological Society, May* 5, 1856.]

THE following notes are devoted to a consideration of the extent to which the human family is affected in the enjoyment of health and the preservation of life, by physical or natural causes. The safest guide in such a field of inquiry is statistics, or the accumulated stores of carefully observed and accurately recorded facts, regarding the occurrence of disease in its different forms, its extension or limitation in space, and the periodicity of its recurrence. But reliable tables of sickness and mortality do not exist, except for very limited and widely separated portions of the globe. In the absence of positive data, however, a knowledge of the physical conformation of the earth's surface, and of the meteorological agencies to which it is exposed, affords a means of arriving at certain probable conclusions regarding others of which little or nothing is known.

The object of Medical Geography is to ascertain the laws by which disease is distributed, or the manner in which certain conditions inimical to the health of the human frame are found to prevail in certain regions or localities. These laws depend for their elucidation on the facts of Physical Geography, embracing geographical position, the nature and elevation of the soil, the amount of moisture, temperature, absolute and seasonal, the direction and force of the winds, and the phenomena of electricity.

In the animal and vegetable kingdoms certain plants and animals are, by natural laws, restricted in their range, within a horizontal and a perpendicular direction, to certain localities ; and in like manner we find that certain classes of disease seldom extend beyond their usual limits, as ascertained by observation, and that they exist and perpetuate themselves only under certain conditions.

The surface of the globe presents, with the utmost diversity of climate and soil, the greatest unity in its pathology. The same morbid appearances are reproduced with the utmost constancy and regularity in a thousand places at once, wherever the same causes of insalubrity are met with, only that they are more intense, continuous, and prolonged, in some places than in others.

Similarity of geological formation indicates a similarity in the diseases of a country as seen in the localities visited by malarial fevers. A certain amount of heat, and a sufficient time for its manifestation, are necessary for the development of certain maladies. In the West Indies, the period of disease follows the course of the sun, the unhealthy season occurring at opposite times on the northern and southern sides of the equator. As the sun proceeds northwards in the ecliptic, so the sickly season advances from the southern to the northern islands. In the Mediterranean the mortality is doubled in the hot season, between July and October ; and in the southern States of North America the posts of the army are regularly abandoned as the hot or sickly season approaches. But in temperate regions the order is reversed. Throughout Europe generally the maximum mortality occurs at the end of winter, and the minimum in the middle of summer. The Registrar-General of England calculates that a fall of the mean temperature of the air from 45° to 4° or 5° below the freezing point, destroys from 300 to 500 of the population of London. The agency of the wind is manifested in the distribution of heat and moisture, and in the comparative density of the air, as well as by its direct influence as a distributor of malarial poison ; and the absence of wind was uniformily noted as a concomitant of cholera, which in

Britain was always most virulent when the calm was greatest, and began to abate when the wind rose. During the great epidemics of Andalusia, 1809-19, the Levanter, a south and south-east wind, was observed to blow constantly for nine months in the year. During the prevalence of the Sirocco, on the Mediterranean shores of Asia and Africa, vaccination fails, as does also inoculation, with small-pox, and ulcers and wounds are more difficult of cure.

As elevation above the surface causes a corresponding reduction in the temperature and in the pressure of the atmosphere, so those diseases which are prevalent at the level of the sea, in cold or temperate countries, are found to be represented by the same, or similar ones, at elevated points in tropical regions, where a corresponding low temperature prevails.

In tropical climates, especially, electricity in its different forms is believed to have a powerful influence on the morbid affections of the human frame; and during the cholera epidemic in Britain, the observations at Greenwich show that the atmosphere was always deficient in positive electricity when the disease was present.

### REGIONS OF DISEASE CORRESPONDING WITH SEASONS AND ZONES OF CLIMATE.

The surface of the globe may be divided into belts or zones, distinguished by great leading characteristics; as I, the torrid zone, or belt of greatest annual mean temperature, characterised by the class of diseases which includes *dysentery, yellow fever, diarrhœa, malarial fevers,* and affections of the *liver*; II, the sub-torrid and temperate zone, of which *inflammatory diseases,* represented by *typhoid fevers,* are the characteristic maladies: and III, the sub-temperate, sub-arctic, and arctic zone, characterised by *catarrhs* and *colds.*

I. The immediate dependence of the first class of diseases on heat and moisture, as important exciting causes, is shown by the circumstance that its maximum intensity corresponds with the countries situated under the line of greatest annual mean temperature, the assumed equator of heat of the globe (82° Fahr.); which line also intersects the region of greatest aqueous deposition. From this line, to about latitude 23° north, 53 per cent of the deaths are attributable to this class of diseases; while in latitude 35° north, marked nearly by the line of 77° Fahr. in July, and on the boundary of the second zone, the amount is only 14 per cent; and at the Cape of Good Hope, latitude 35° south, it is only 3 per cent.

As far as can be ascertained, the mortality from the entire classes within this zone amounts to 75 per cent,—the first and second causing 53, and the third 18 per cent. of the whole. The same law of decrease with the lowering of temperature is apparent in the seasons of their occurrence. In a series of dysentery epidemics, narrated by Ozanan, 36 occurred at the end of summer, 12 in autumn, and only 1 winter. Of 13,900 individuals seized with dysentery in Bengal, 7,000 were attacked in the warm and humid season, 4,500 during the hot and dry season, and 2,400 during the cold season. In spring these diseases are more inflammatory in their character, and in autumn more putrid.

The northern limit of this class of diseases is probably the Bermudas, latitude 32° north, in the Atlantic; and California, latitude 38° on the Pacific Ocean, in America. In Asia it extends to near Pekin, latitude 40° north; and in Europe to the south of Spain. Its southern limits are—in America, Buenos Ayres, latitude 34° south, on the Atlantic, where, however, it is not severe; and Lima, latitude 12° south, on the Pacific. In Asia the southern limit includes Aracan, Ava, and Ceylon, Borneo, and the other islands of the Asiatic Archipelago, and thence it extends to the northern shores of Australia. In Africa it includes the island of Madagascar. Within these limits the principal centres of these diseases are, in America, the shores of the Gulf of Mexico, the West India Islands, and the northern portion of South America; in Asia—India, China, Borneo, Ceylon; in Africa—the countries around the Gulf of Guinea on the west, Madagascar and Mozambique on the east, Algeria and the shores and islands of the Mediterranean on the north. Little is known of the perpendicular distribution of these diseases, except that in Mexico they are prevalent at an elevation of 7,000 or 8,000 feet; and in south-eastern Asia they cease at an elevation of 6,000 or 7,000 feet above the sea.

II. In the inflammatory region, or zone, typhus fever, in its varied forms of gastric, bilious, enteric, &c., fever, takes the place of the yellow and malarial fevers of the torrid zone; and in consequence of fewer of the population being cut off with these, more fall victims to inflammatory affections, of which consumption is the type. But that this latter form of disease is not peculiar to this region, or rather that it becomes more fatal as we approach the tropics, is proved by the fact that in England consumption is only fatal to 3.8 out of every thousand living, while Boston (U.S.) loses 4.0, Baltimore 4.1, Philadelphia 4.2, New York 4.9, and New Orleans 5.6 out of every thousand living.

In North America and Europe, the southern boundary of this group of diseases coincides generally with the northern boundary of the first class. In South America, it probably includes Patagonia. In Africa, it includes the Cape Colony; and it embraces the South of Australia, Tasmania, and New Zealand. In Asia it is uncertain how far it extends to the eastward. Its northern limit in America includes part of Nova Scotia and Newfoundland; and in Europe the northern boundary includes the British Islands, Norway and Sweden, to latitude 60° north, whence it appears to follow a south-eastern direction, corresponding nearly with the annual isotherm of 41°, till it gradually declines towards the borders of Asiatic Russia. These, however, are only to be considered as preliminary indications.

III. The boundaries of this group of diseases, which is characterised by catarrhs, include the whole of Europe to the north of the preceding class. In America it extends south to Boston and New York, including the district of the Canadian Lakes. Thence it continues north-west nearly on the line of 41° annual temperature. Although very little is known of the diseases of Central Asia, yet, when we consider the elevation of the surface, the vegetation and the conditions of climate, we may assume that this class of diseases extends there to about latitude 45°. Iceland is the best-known locality of this zone, and may therefore be taken as its representative. The island is visited by catarrh every year in spring or in early summer. It is also visited at short intervals by catarrhal fevers,—a true influenza, which usually has a great effect on the mortality. Pallas says that the majority of Icelanders die, before the age of fifty, from asthmatic or catarrhal affections of the lungs; and Crantz affirms that catarrh is a very prevalent disease in Greenland. Catarrh is also common in Labrador. At Okhotsk in Siberia it is accompanied with difficulty of breathing; and a cough, called "Ho," is endemic among the Samoeids.

### SKETCH OF THE CLIMATOLOGY AND DISEASES OF THE DIFFERENT QUARTERS OF THE GLOBE.

EUROPE.—The continental portion of Europe presents the greatest contrasts in its climate, but it is generally temperate, owing to the extent of sea on its coasts, its numerous inland lakes and rivers, and the Gulf Stream of the Atlantic, the heated atmosphere of which is borne to its shores by the prevailing south-westerly winds. South of latitude 45°, extreme cold is rare and of short duration, and the heat due to its position is tempered by the elevation of its mountains; but the southern coast-lands are blighted by the hot wind of Africa, the *sirocco;* and, from its exposure to the northerly winds from the Arctic Ocean, the great north-east plain has a severe cold climate.

Nearly every form of disease has its representative in Europe; especially cretinism and goître in the Alps, Caucasus, and Urals, the high lands of Bosnia, and the mountains of Scandinavia; typhus between the parallels of 44° and 60° in Western Europe; yellow fever occasionally on the shores of Spain and Northern Italy; intermittent fever in the Netherlands, a portion of Sweden, Central Italy—in short, wherever marshes exist; consumption in all parts; the plague in the eastern countries; small-pox especially where vaccination has not been introduced; leprosy and elephantiasis in Scandinavia; pellagra in Italy, France, and Spain; and plica Polonica in Poland and Tartary.

ASIA.—The peninsula of Western Asia, from its peculiar formation and the elevation of its table-land, presents striking anomalies in its climatology and corresponding varieties in its temperature. The plague extends occasionally between the parallels before mentioned to the borders of Persia. Remittent fever prevails along both shores of the Red Sea and the Persian Gulf, but is not severe, except in the marshy districts at the mouths of large rivers; and dysenteric affections are seldom met with in either of these regions.

The chief physical features of *Persia* are its vast central table-land, 2500 to 3500 feet above the sea, and the wide salt desert occupying its eastern provinces. South of the table-land, the country is parched and barren, and the heats of summer are almost insupportable. This country, like Beloochistan, suffers from a scarcity of water; but both are comparatively healthy, and not the seat of any remarkable disease. In Bokhara, intermittent fevers are prevalent at the end of August and beginning of September; they disappear with the first frost. In *Tibet,* small-pox is extremely dreaded, and the infected house or village is razed to the ground. Inoculation is practised in *China,* but not in Tibet. The other complaints are dropsy and liver disease; fevers are seldom fatal.

*India* has every variety of surface, from the level of the sea to the highest mountains on the globe, and its climate partakes of all changes. The year has three seasons—hot from March till June, rainy from June to October, and temperate from October till the end of February. The monsoons regulate the hot and dry seasons; earthquakes and violent hail-storms are of frequent occurrence, and often cause great destruction to human life. The inter-tropical portion of India comprises the stations of Calcutta, Madras, and Bombay, with an area nearly equal to the northern extra-tropical portion. In Bengal the climate is hot and humid from April to November, the other months are cool and bracing. At Calcutta the mean annual temperature is 90°, and the fall of rain 64 inches. The rainy and stormy season is during the south-west monsoon, from June to September. Madras has a mean annual temperature of 83°, and a mean rain-fall of 51 inches. The north-east monsoon brings thunder and rain, but the country is sheltered from the south-west monsoon by the range of the Ghauts. In the hot season the cool sea-breeze called "the doctor" blows from noon to nightfall, and is followed by the sultry and oppressive land-wind, which prevails till noon of the following day. In April and May the south shore-wind produces severe rheumatism. At Bombay the mean temperature is 85°; it is seldom above 100° or below 70°; the mean rain-fall is from 66 to 80 inches. At Delhi, 800 feet above the sea, the climate is dry, the rain-fall being only 20 inches. In the valley of Cashmere, 5000 feet above the sea, there is frost and snow in winter, and in July and August the thermometer rises to 80° or 85° at noon. At the Sanitarium of Darjeeling, 7500 feet above the sea, the mean annual temperature is about 50°—nearly that of London—and the rain-fall is 125 inches.

The diseases most prevalent in the lower districts are, among Europeans, dysentery, liver affections, fevers, and rheumatism; and among the natives, leprosy, elephantiasis, Guinea-worm, ophthalmia, and beriberi. On the delta of the Indus, and along the western and eastern shores of India, remittent fevers and dysentery are the chief diseases of Europeans; and from the valley of the Hoogley, along the shores of Aracan and Rangoon, they are endemic, and of frequent occurrence. Influenza is often very severe, and cholera comes every year in the delta of the Ganges towards the end of the hot season. Small-pox formerly committed great havoc, but of late the virulence of the disease has been checked by inoculation. Europeans have found great benefit from a temporary residence at the different Sanitaria established at elevated stations in the sub-Himalayas, the Neilgherries, &c. Darjeeling affords instant relief from acute diseases; cholera is hardly known there, and when imported it does not spread: liver and bowel diseases are equally rare; and ophthalmia, elephantiasis, and leprosy are almost never seen; but rheumatism and ague are frequent, as well as violent and often fatal remittents.

The hill-countries of Gurwhal and Kumaon, at the foot of the Snowy Mountains, are visited by a pestilent disease termed "*Maha Murree*," or "certain death," which is described as resembling the plague of Turkey, and as being so infectious that any one afflicted with it who dared to leave his village or hut was shot like a mad dog. It commences with violent pains, succeeded by swelling of the body: it is generally fatal in 24 hours and is said to cut off 99 of every 100 attacked. Goître is prevalent in the valleys of the Himalaya, in the mountainous countries of Central Asia, and in the island of Sumatra. Diseases of the liver and bowels cause one-half of all the deaths among Europeans in India; fevers are slight. Liver disease is almost unknown among the indigenous population; and consumption, which is slight among Europeans, is scarcely ever met with among the natives. The average mortality among British troops for all India is 57 per 1000, and for native troops 18 per 1000, or less than one-third.

The island of *Ceylon* has a hot and moist climate; on the sea-coast the temperature ranges from 68° to 90°,—the mean being from 75° to 80°. The north-east monsoon prevails from November to February, and the south-west from April to November, and any interruption of their regular courses greatly increases disease. The east part of the island is hot and dry, the west more temperate and humid. The rain-fall is 85 inches at Colombo, and 120 inches in the hilly districts. The diseases of Ceylon resemble those of India. Small-pox is always more or less prevalent, and ophthalmia is common in the dry season. The army-returns show a mortality among the troops of 75 per 1000, or five times more than in Britain.

The climate of *Burmah* is comparatively healthy, but that of Aracan proved most destructive to the British troops in the campaign of 1824-1826, when in a short period from one-half to two-thirds perished. The chief maladies are fever, disease of the digestive organs, and cholera; consumption is said to be rare.

*Malacca* has an equable healthy climate—the thermometer ranging from 72° to 85°; it is but little affected by the monsoons, but there are regular land and sea breezes. The principal diseases are remittent fevers, with occasional outbreaks of cholera. Singapore is remarkably free from the diseases of the surrounding countries. *Sumatra* has mountain chains rising to 15,000 feet above the sea, but its eastern portion is level or undulating, with marshy plains along the shores: temperature at mid-day 82° to 85°, but at sun-rise not more than 70°. Thick fogs, storms, and water-spouts are frequent off the coasts. *Java* is traversed by a mountain chain from west to east, about 1000 feet in elevation, with volcanic peaks rising to 10,000 feet: its north coast is low and marshy, and is lined with numerous small islands. The mean annual temperature at Batavia is 78.3°; winter, 78.1°; summer, 78.6°: at mid-day it rises to 80° or 90°, and at night it falls to 70°. Remittent fevers, dysentery, and cholera, are the principal diseases.

*Borneo* has low swamps and paddy-fields on the western shores, where dysentery prevails; the other diseases are remittent fevers and cholera, which latter are the diseases peculiar to the Moluccas; but Celebes, from its singular formation exposing it to every wind, is very healthy.

The *Philippines* have a hot moist climate, and are subject to severe storms. Winter, during the north-east monsoon, is the most healthy season. The mean annual rain-fall is 98 inches. The natives are healthy, and longevity is common. The principal diseases are intermittent fevers in low situations, chronic dysentery, elephantiasis, leprosy, and the *berba,* a disease characterised by swelling of the abdomen. Cholera was epidemic in 1842. Disease of the lungs is rare.

The coast of *China,* between the parallels of 20° and 40° north latitude, is most unhealthy, especially during summer and autumn, presenting throughout a humid atmosphere, a marshy soil, and a rank vegetation. The most peculiar complaints are dysentery and intermittent fevers, ending in remittent fevers, and sometimes complicated with scurvy. Intestinal worms are also very common. The small islands adjacent to the coast are equally unhealthy, and the diseases are of a similar kind. Yellow fever has not yet been observed, but cholera has been severe on the coasts, and in the islands of Amoy, Hong-Kong, Chusan, and at Manilla.

*New Guinea, Australia,* and *Tasmania,* are exceedingly healthy, and cholera has not yet appeared. New Zealand, as well as all the islands of the Polynesian Archipelago, are remarkably free from the more fatal maladies which infest the shores of Asia and Africa. The climate of Australia presents great variety. The mean temperature of spring is 72°, autumn 66°, winter 55°. In Sydney the thermometer is seldom below 40°, but at Paramatta it is frequently 27° in winter. At Adelaide, South Australia, it ranges from 48° in July to 101° in January, and at Melbourne from 54° in June to 73° in January. The air is very elastic. The hot winds, supposed to originate in the deserts of the interior, raise the thermometer in the shade to 117° or 120°; they wither the grass, and frequently destroy the harvest, but do not appear to affect the health of man. The diseases of Australia resemble those of Britain, but they assume a milder type; the principal are those of the alimentary canal, the respiratory organs, the brain and nerves, and rheumatism. Dysentery is occasionally severe in South Australia, Victoria, in the Digging regions pre-eminently, and Moreton Bay; but not in Sydney. The only part of Australia at which remittent fever has been known to prevail is Port Essington, on the north coast. Typhus has been introduced by ships, but it loses its virulence. Ophthalmia is common in South Australia, and in 1847-8 epidemic influenza was fatal to European children and the aged, and cut off 15 per cent of the natives. Pulmonary consumption is scarcely known among the aborigines, but small-pox is prevalent. The mean temperature of Tasmania is about 70°, but during the hot winds from the north and north-west, it rises to 100° or 110°; the minimum is 31° in July. In the winter months, June, July, and August, frosts are occasionally severe in the high lands of the interior. The average number of rainy days is from 100 to 120, and the rain-fall 23 inches. The diseases resemble those of Australia, but the island is generally very healthy.

*New Zealand.* At the time of Cook's first visit, the islands were remarkably healthy, but since their intercourse with Europeans the natives have rapidly declined. In 1848 the deaths of Europeans in the hospitals amounted to only about 9½ per 1000. Catarrhal disease was very prevalent in 1849. Influenza, scarlatina, consumption, and scrofula have made great ravages among the natives, and rheumatism is also prevalent among them, but these maladies are rare and mild among the English. The islands are almost exempt from remittent and intermittent fevers, Epidemic scarlatina of a fatal type appeared for the first time in 1848. Small-pox and measles have not yet visited New Zealand, and vaccination is extensively practised. A strange disease called "Ngerengere" (*lepra gangrænosa*), formerly prevalent among the New Zealanders, is now rare.

Africa, in so far as is known, comprises the most healthy and the most deadly climates on the globe—the former at its southern extremity, and the latter on the west coast.

*Algeria*, on the north coast, is traversed by the Atlas Mountains, which rise to 7000 feet above the sea; on their northern slopes the climate is temperate and healthy, but it is pestilential in the marshy plains. The heat is often excessive under the influence of the *simoom*, or hot wind of the desert. In 1846 there were fifty-six storms. The most fatal maladies are diarrhœa, dysentery, and liver disease.

On the *west coast* of Africa the shores and estuaries of rivers are low and marshy: the chief characteristic of the climate is excessive moisture, the average annual fall of rain at Sierra Leone being 189 inches, and the mean temperature 81°. The rainy season extends from June to September. After the rains, dense masses of vapour termed the "smokes," envelope the land for days together, and are often driven to a considerable distance seaward. The temperature of the inter-tropical portion, from the Gambia on the north to Benguela on the south of the equator, is remarkably equable, the general range of the hottest period being from 80° to 86° in the shade on board ship, while at the extremities of the station in the winter months it is seldom below 58°; but sudden changes from excessive heat to cold, with chilling fogs, are frequent. Every part of this coast, and the adjacent islands between the tropics, is most deleterious to the health of Europeans, but much of the excessive mortality recorded there has been the result of imprudence.

The most fatal climatal diseases are remittent fever and dysentery. July to October, the rainy season, is the most unhealthy; from November to April fever is comparatively mild. Dysentery is most prevalent, both on shore and in ships, in the southern division of the station, probably owing to the use of the waters of the Congo river. It is frequent in all seasons at Ascension, but is not endemic at Sierra Leone. Inflammation of the liver is less prevalent here than in India, and consumption is little known. Among the natives, especially in slave-ships, the prevailing diseases are dysentery, fever, small-pox, rheumatism, lethargus, or "sleepy dropsy," and ophthalmia. Of the white troops who garrisoned the stations at Sierra Leone in 1824, two-thirds died in a year, and few lived to complete twelve months in the command. The annual mortality among the black troops is only from 2 to 3 per cent. The Guinea worm infests the negroes, or others who have resided some time on the Gold Coast, or on the shores of the Bights of Benin and Biafra. Yaws and craw-craws are also prevalent among the natives.

The climate of *Loango* is fatal to Europeans; its chief maladies are remittent and intermittent fevers. St. Paul de Loando has a climate intensely hot; fevers prevail, and ague and small-pox occur among the natives. Benguela is reported the most sickly of the Portuguese stations south of the equator: the heat is here excessive, and water deficient. The southern portion of Africa, beyond the tropic on both sides, is remarkably free from remittent fevers.

In the *Cape Colony* the climate may be termed temperate, the mean temperature is 67°, and the range is from 50° to 80°. Sudden changes are common, a veering of the wind from north-west to south-east often lowering the temperature 40° in one day: and the hot winds in May, June, and July, frequently blow with the violence of a gale, and raise the temperature 20° in course of a night; but this apparently does not injure health. The country is eminently healthy, and cannot be said to have any peculiar diseases. Dysentery sometimes attacks new immigrants, and many of the Hottentots die of consumption, and present cases of leprosy. Diseases of the stomach and bowels are general, and rheumatism is severe and very prevalent; but fevers are of rare occurrence.

In *Kaffraria* and *Natal* winter is the dry season. From May to August it seldom rains: in summer the rainy season sets in with terrific thunderstorms. In spring the temperature of the plains is seldom above 50°, in summer it is between 70° and 90°, and before storms it often rises to upwards of 100°. The country is remarkably healthy.

*Madagascar* is visited by pestilential fevers and dysentery on the west coast, and by aggravated skin-diseases in the interior.

*Mozambique* has long been notorious for its pestilential diseases. From Delagoa Bay to Magadoxo remittent fever and dysentery prevail at all seasons along the coasts of Zanguebar, and extend a considerable way inland, but the more elevated portions of the country of the Imaun of Muscat are said to be healthy.

From the equator to the Strait of Bab-el-Mandeb, and along both shores of the Red Sea, remittent fever prevails in the marshy districts, at the mouths of large rivers. Remittent and intermittent fevers, dysentery and ophthalmia, are prevalent in Abyssinia.

*Egypt*. The climate of the upper part of the Nile Valley is characterised by extreme dryness. The temperate season lasts from October to March, and the hot season from March to September. In summer, the heat during the day is excessive, owing to the confined position of the country and the low level of its surface, but the nights are cool and agreeable. In winter the weather is mild and pleasant. At Cairo the mean temperature of the year is 72°.2, winter 58°.4, summer 85°.1; and at Kenneh the mean is 79°.9, winter 63°6, summer 92°. Upper and Middle Egypt are more healthy than the Delta. The annual inundation of the Nile commences in June, and attains its maximum height in September. After remaining stationary for some days, the waters again subside, when fevers, dysentery, and ophthalmia prevail over the whole country. The plague is endemic in the lower province, seldom passing south of Siout, on the Nile. North and north-west winds blow permanently during the progress of the sun towards the tropic of Cancer; but on his return to the tropic of Capricorn, it varies between south-east and west. At the spring equinox the pestilential hot wind, called the *Simoom* or *Khamsin,* blows from the south-south-west for fifty days. During this period the diseases peculiar to the country assume their greatest virulence. The *mirage* occurs on the extensive plains after the surface has been heated by the sun; the country then appears like a vast lake studded with islands. Rain is unknown in Upper Egypt; in the Delta it falls frequently from November to March. Showers are slight and infrequent at Cairo; the average number of rainy days there being thirteen in a year. Snow never falls except in the vicinity of the coast, and then in very small quantities.

AMERICA, NORTH.—The great characteristic feature of this division of the so-called New World is its vast interior valley, extending from the Gulf of Mexico to the Arctic Ocean, and presenting every variety of climate between the tropical and the polar regions. This valley is cut off from the genial influences of the Pacific Ocean on the west by the Rocky Mountains, and from those of the Atlantic Ocean on the east, but in a less degree, by the Alleghanies. It is traversed by a deep winding longitudinal depression, forming the trough of the Mississippi for more than 2000 miles. The climate of the north-eastern States is variable, with extremes of summer heat and winter cold, while the southern States enjoy more of a tropical climate. The Pacific coasts are milder, and in the north more moist than those of the Atlantic. The following examples show the decrease of temperature and rain-fall in proceeding from south to north:—

| Places. | Latitude. | Temp. of Year. | Annual Rain in inches. |
|---|---|---|---|
| New Orleans | 29·57 | 71·32 | 52 |
| Cincinnati | 39·06 | 54·25 | 47 |
| Washington | 40·22 | 53·13 | 34 |

Throughout the eastern half of North America, especially its middle latitudes, there are but three characteristic winds:—1. A damp and chill current from the north-east, visiting the whole Atlantic sea-board and the interior to about lat. 35°;—2. A south-west wind, extending from the Gulf of Mexico and the table-lands of the continent, the whole way to the basin of the St. Lawrence, and even past it; and—3. A continental wind from the north-west, dry and cool, and very pure. The north-east wind blows on the fewest number of days in the year, except upon the north-east Atlantic coast, where it is very frequent. In the southern half of the country the south-west wind predominates, especially in summer and autumn; and in the northern half the north-west wind is in excess, particularly during winter and the latter part of autumn. Of the relations of these winds to disease, it may be stated generally, that the north-east is the *catarrhal* wind, disarranging most the respiratory organs; the south-west wind the *malarial* wind, disturbing most the functions of the liver; and the cool, dry, continental wind from the north-west is by far the most salubrious breeze on the continent, tending to repair the mischief done by the others, especially by the south-west.

In Louisiana the most fatal disease is the congestive form of fever, called the *cold plague.* Diarrhœa and dysentery prevail extensively, mostly south of the 40th parallel of latitude, and the dengue, "breakbone" or "dandy" fever, is prevalent as an epidemic in the southern states. The same fever was observed at Rangoon and Calcutta in 1824, at Burhampore, etc., in 1825; the island of St. Thomas, West Indies, in 1827; and New Orleans in 1828. A remarkable endemic disease, termed the *milk sickness,* is peculiar to the western portion of the United States, being seldom or never observed to the east of the Alleghanies.

The mainland of North America, from the tropic of Cancer to Behring Strait on the Pacific side, is free from endemic diseases, but yellow fever has of late years appeared on the south-west coasts. From the parallel of 46° or 47° north to the polar regions, the inhabitants, with the exception of exanthematous diseases (eruptive fevers or rashes) and influenza, enjoy the most perfect immunity from the endemics and epidemics which infest the tropical regions. The Esquimaux have few ailments, and typhus fever and consumption are all but unknown in the arctic regions. Cholera first appeared in the ports of the United States in 1832.

*Oregon* has a climate well suited to the white race. The western section is mild and equable, resembling that of England; south and south-west winds prevail on the coast; the middle section is dry and changeable, the temperature varying from 9° in January to 108° in July. In the eastern section, the temperature fluctuates 50° or 60° in a few hours. No ague nor fevers were known to exist prior to 1830, but since then they have committed frightful ravages amongst the Indian population.

*California* is much milder in its climate than the corresponding countries on the Atlantic side, with short winters. At San Francisco the temperature is seldom above 80°, and rarely falls below 40° in the rainy season. Fogs are frequent in summer, from north-west winds. Snow is seldom seen on the coast. The sheltered valleys enjoy an excellent climate. In the valleys of the Sacramento and San Joaquim, the mercury often rises to 100° or 120°, and the air is extremely dry. The white population is exempt from climatic diseases, but in 1839 small-pox carried off one-half of the aborigines.

*Mexico* is traversed by the mountain chains which connect the Andes and the Rocky Mountains. These separate and enclose the table-land of Anahuac, 6000 to 8000 feet above the sea. The *Tierras Calientes* (hot lands) extend from the coast to about 900 feet above the sea; *Tierras Templadas* (temperate), 4000 to 5000 feet; and *Tierras Frias* (cold) above 7000 feet. The mean temperature of the coasts between the parallels of 15° and 20° is 76°, while on the elevated plains it is only 64°. The shores, especially those of the Gulf of Mexico, are excessively hot, humid, and unhealthy; while the plains of the interior, 3500 to 4500 feet high, have a temperate and perfectly healthy climate. The annual rain-fall at Vera Cruz is 185 inches. All the higher regions of Mexico are extremely healthy; fevers are confined to the coasts. The *Matlazuahuatl* is a disease peculiar to the Indians; it resembles yellow fever. Small-pox was introduced into Mexico in 1520, when it destroyed one-half the population. In 1799 it destroyed in the city of Mexico alone upwards of 9000 persons.

The state of *New Mexico* is extremely salubrious. The annual range of temperature is from 10° to 75°, and the rainy season from July to October. Bilious diseases, the scourge of the Mississippi valley, are almost unknown here, but an epidemic fever, of a typhoid character, ravaged the country in 1848-9; and this, together with small-pox, which prevailed epidemically in 1840, carried off 10 per cent. of the population.

In *Central America* the climate is remarkable for its variety of temperature produced by difference of altitude, and its equality during the different seasons of the year. On the south-west coast the rainy season begins in May and ends in October. During the rest of the year rain is almost unknown. On the north-east coast the rains continue nearly all the year, the driest period being from June to October, and the wettest from October to May. The excessive moisture renders the north-east coast very unhealthy, while the rest of the republic is, considering its position, comparatively salubrious. On the table-lands (*los Altos*) the temperature is mild, and in the higher stations it is excessively cold. In Guatemala the temperature is seldom above 80° or below 50°. Many of the largest towns, as Sonsonate and San Miguel, being little above the level of the sea, have an oppressively hot temperature of 80° or 90° at all seasons. Honduras has a very unequal surface, and the capital town, Comayagua, has a hot climate; but many parts of the interior have a fine temperate climate, similar to that of Southern Europe. Belize is reckoned more healthy than most of the West India islands: the mean annual temperature is 80°: rains are frequent in July, August, and September. Omoa and Truxillo, on the north-east coast, have an excessively hot and unhealthy climate. Yellow fever occurs on all the coasts of the Mexican Gulf, and goître is prevalent in the mountainous districts. At Porto Bello the "vomito negro" has often nearly depopulated the place.

*West India Islands.* The space between the equator and the parallel of 40° north, including the shores of the Caribbean Sea, the Gulf of Mexico, and part of the Atlantic shores of the United States, with all the West India islands, is the true domain of yellow fever. Remittent fevers also, the endemic of hot countries, and dysentery, prevail at all seasons, occurring most frequently in marshy places. This is, besides, the region of hurricanes, and the theatre of many epidemic diseases. But although all the islands are included within the pestilential limits, they present a great variety in their comparative liability to and exemption from particular forms of disease.

*Cuba.* The climate is changeable and cold during N.N.W. winds. The hottest months are July and August, when the temperature is 82° or 84°. In the coolest months, December and January, it falls to a mean of 63°. The average of rainy days at Havana is 102. The mean annual fall of rain is 45 inches. Hurricanes recur every second year, in September and October. The towns on the coast are inimical to the health both of the black and white population: the western portion of the island is the most unhealthy, and the central least so. The greatest mortality occurs between the years of twenty and thirty, the age at which the greatest number of immigrants arrive. The most fatal months for the Creoles are March, February, January; the least so, November, December, June. The most fatal months for Europeans are June, July, August, May; and the least so, January, February, March, November, and April. Intermittent fever prevails along the rivers of Cuba, but yellow fever does not extend to the interior.

*Porto Rico* has a climate generally more healthy than the other islands of the Antilles. Cholera broke out in December 1855, and extended over eight towns.

In *Jamaica* the climate varies greatly at different elevations. At Kingston the mean annual temperature is 80°, while at Pleasant Hill, 4000 feet above the sea, it is from 52° to 65°. The amount of rain at some seasons is very great, varying from 70 to 100 inches. In the mountains the climate is peculiarly healthy, and they are now a favourite resort for American invalids. The chief diseases are yellow fever, remittent and intermittent fevers, diarrhœa, dysentery, rheumatism, and influenza. The negroes suffer from ulcers and *yaws*, a species of leprosy. Cholera appeared for the first time in October 1850, at Port-Royal, thence it spread over different parts of the island. It is calculated that in 1850-51, a fifth part of the population was attacked with the severe form of the disease, and the deaths are estimated at from 30,000 to 50,000. Yellow fever, in its most virulent form, appears very frequently at Port-Royal.

*Barbadoes.*—Climate equable, and comparatively dry; temperature from 77° to 84° Fahr.; annual rain-fall 72 inches; prevailing wind north-east. The island is subject to terrible hurricanes and earthquakes; thunderstorms are frequent and severe. It, as well as Antigua, is frequently visited by yellow fever of a severe type.

*St. Lucia.*—Temperature of the year from 75° to 90° Fahr.; annual rain-fall, 84 inches. The island has long been noted for its insalubrity, the chief diseases being fevers, and diseases of the stomach and bowels.

*St. Kitt's* is extremely dry, and comparatively healthy.

*Tobago.*—The climate of this island is healthy, on account of its narrowness, and the regularity of the land and sea breezes. The thermometer ranges from 75° to 90°. Fever is prevalent.

*Trinidad* is in general healthy, but fevers are severe. The thermometer ranges from 70° to 85°, and the fall of rain is 75 inches. The hot and rainy season extends from June to October, but hurricanes are unknown.

*Bahamas.*—The climate is equable, the temperature varying from 73° to 93° Fahr. The islands are resorted to by Americans afflicted with pulmonary complaints. Cholera has never visited the Bahamas.

The *Bermudas* are generally healthy; the thermometer from 60° to 70° Fahr. In 1842 epidemic influenza raged with great violence among the white population, but the blacks were very slighly affected. Rheumatism is prevalent, and dysentery and yellow fever prevail occasionally. Remittent fever is not severe.

*Canada.*—The climate of this vast country varies according to position and elevation. It is everywhere liable to sudden changes. In the eastern province the winter is more severe than in the west, but the clear blue sky, and the absence of fogs, indicate its great salubrity. The mean annual temperature of West Canada is 48.37°; and of East Canada 42.1°. The annual fall of rain is nearly the same as on the east coasts of Great Britain. At Quebec the mean winter temperature is 14.2° Fahr.; but it is sometimes as low as 60° below the freezing-point. The prevailing winds are westerly. The severest winters are accompanied by north-east winds. The heat of summer is less relaxing, and the cold of winter more bracing, than in the United States. As the country becomes cleared, and has its swamps drained, its inhabitants may hope to enjoy a climate as salubrious as that of Britain. During the *Indian summer*, in November, the temperature is mild and serene, with a hazy atmosphere. The principal diseases are ague and typhus fever; the former most prevalent in Western and the latter in Eastern Canada.

*Newfoundland.*—The climate is less severe than in Canada; the frost is less intense, and snow does not lie long on the ground. It is subject to dense fogs, chiefly in May and June, but these do not appear to injure the health, and the climate is remarkably salubrious. The chief diseases are those of the lungs, catarrhs, and phthisis; next, those of the liver and of the stomach and bowels, fevers being small in proportion. The mortality, according to population returns, is only 1 in 76; but the return of the troops in the station is much less favourable.

*Nova Scotia.*—Climate very moist; fogs are common along the coasts in May and June; changes of temperature sudden and extreme; range of thermometer, 6° or 8° below zero in winter, to 88° in summer. The air is highly salubrious; the inhabitants enjoy a remarkable degree of health, and an almost total exemption from the intermittent and remittent fevers of Canada and the United States.

*Cape Breton* has a similar climate, and is even more healthy, no epidemic disease, except small-pox, having been known for many years previous to 1834.

*Prince Edward Island* has a more severe winter, the thermometer being often 20° to 25° below zero; and the rivers and bays are frozen till the end of April. The army returns show a mortality of 14 per 1000, the chief maladies registered being diseases of the lungs, fevers, and rheumatic affections. Epidemic cholera did not visit Nova Scotia or New Brunswick in 1832, when it raged in Canada, but it appeared at Halifax in July 1834. It did not extend beyond the limits of the town. In 1849 the course of cholera in British North America was nearly the same as in 1834.

South America, from its peculiar formation, having a girdle of lofty mountains along its western side, and no corresponding continuous chain in the east, discharges nearly all its waters eastwards to the Atlantic Ocean, through the great basins of the Orinoco, the Amazon, and the Rio de la Plata. In general the climate of South America is colder, and more moist than the corresponding portion of the Old World. Among the causes which produce this effect, Humboldt assigns the extension of the narrow part of the Continent towards the south pole; the expanse of ocean over which the trade-winds blow; the flatness of the eastern shores, and the current of cold water on its western coasts; the lofty mountain-chains, whose snow-clad summits cause currents of cold air to roll down their declivities; the numerous large rivers, which, after many windings, seek the most distant shores; the grassy steppes, which are not susceptible of acquiring a high temperature; and the impenetrable forests, near the equator, screening the alluvial soil from the direct rays of the sun, and exhaling vast quantities of moisture.

*Venezuela* has a hot dry climate along the shores of the Caribbean Sea, but Caraccas, 2,822 feet above its level, is healthy. The table-land near the coast has an annual temperature ranging only from 70° to 80°. In the Llanos, or plains, the climate is unhealthy.

*British Guiana* is excessively moist. In 1830 it rained continuously, except during September and October. In 1831 the rain-fall at George Town was 157 inches. The mean annual temperature is 81°, max. 90°, min. 75°; nearly the same in Demarara and Berbice. It is not subject to hurricanes. The most unhealthy season is from June to October. The chief maladies are intermittent and yellow fevers, stomach and bowel complaints, and disease of the lungs. Spleen diseases are common as a consequence of intermittent fever, especially among immigrants. Yaws, an African malady, common during the time of slavery, has almost entirely disappeared, and the same may be said of leprosy. Elephantiasis is very common among the coloured population.

*Brazil* has, on the whole, a mild and healthy climate. The rainy season commences with thunder-storms in October, and lasts till March. The mean temperature at Rio is 72°. The clearing of forests here has reduced the amount of rain so as to render the water supply deficient. Para, formerly remarkably healthy, and free from epidemics of any kind, was visited by yellow fever in February 1850: its period of greatest malignity was during April, when the deaths in the city were twenty to twenty-five per day. About the same time in the following year, yellow fever having greatly abated, small-pox broke out with great violence; and by these two diseases about 25 per cent. of the population was carried off. Cholera appeared for the first time at Para in May 1855, it reached Bahia in the middle of July, Rio de Janeiro on the 20th of the same month, and Pernambuco in February 1856. At Rio the whites died at the rate of 1 in 126 of the population, while among coloured people the proportion was 1 in 52. The climate of Para is delightful, but its filthy condition is a fertile source of malaria. At Santarem, in the province Para, elephantiasis and leprosy are common among the poorer classes. Bahia was long celebrated for its general health, and its freedom from cholera, influenza, and dysentery; but it also was visited by yellow fever for the first time in November 1849, although a similar epidemic is said to have been observed at Bahia and Pernambuco, between 1612 and 1686. A species of typhus, termed *febres malignas,* commencing as ague or remittent fever, is prevalent on the coasts of Brazil, and cretinism and goître are common in the mountain valleys of the interior. Elephantiasis, or the "Barbadoes leg", is still frequently met with, although it is decreasing among the white population; and the frightful malady termed *elephantiasis Græcorum,* or tubercular elephantiasis, a species of leprosy, is common at Bahia, where there is a hospital for lepers. Insanity, nervous and pulmonary diseases, formerly rare, are greatly on the increase since the independence of Brazil. Hepatitis, though not so general as in other hot countries, is also on the increase.

*Paraguay* is described as generally very healthy, its tropical heat being modified by the inequality of its surface; yet it is occasionally ravaged by the most fatal maladies. In ten years previous to 1840, 20,000 persons died of dysentery; from 1836 to 1838, 11,000 died of scarlet fever; and in 1844-5, 14,000 died of small-pox, in a population of about 200,000.

*Buenos Ayres* has a climate esteemed one of the healthiest in the world, and instances of longevity are common. The *pampero*, or south-west wind, is often a hurricane, bringing clouds of dust from the parched pampas, and causing complete darkness, which lasts for half an hour in the middle of the day; and the *viento norte* (north wind), is very severe, producing great irritability and headache, chiefly among the native women. Small-pox formerly cut off thousands of Indians, and its ravages are still very severe among them; but it has been greatly arrested by vaccination. Intermittent fever is hardly known. The most fatal diseases in the hospital in 1828 were consumption, fever, and inflammation of the liver.

At the *Falkland Islands* the climate is more equable than in England; the temperature ranges from 30° to 50° in winter, and from 50° to 75° in summer; snow seldom lies on the ground more than half an inch thick; rain is frequent, but mild. The prevailing winds are north-west in summer, south-west in winter. The islands are very healthy; no peculiar disease has appeared, and residents afflicted with pulmonary complaints are said to experience relief.

On the west or Pacific coast of South America, remittent fever, but not of a severe type, extends from Panama to near Lima, latitude 12.3° south, and yellow fever has appeared on the coast as an epidemic several times; but the Galapagos Islands, near the centre of this district, have escaped these maladies, and are remarkably healthy.

*New Granada* has an extremely varied climate; at Honda, 1000 feet above the sea, the heat is intense. Mompox has a dense moist atmosphere, and goître and malignant ulcers prevail. At Cartagena the yellow fever is endemic, but in the elevated regions of the Andes the air is highly salubrious, with a temperature of 55° to 70°. On the coasts of Ecuador at Guayaquil, the country is inundated during the rainy season in July, after which it remains for some months a pestilential marsh; here yellow fever has appeared several times. At Quito, 9,543 feet above the sea, there is perpetual spring, with a mild and equable and healthy temperature, but it is disturbed occasionally by violent winds and devastating earthquakes.

*Peru* has a hot and dry climate on the Pacific slope, where rain never falls, but it is tempered by the numerous streams which descend from the mountains, by the "garua" fog, and by the current of cold water from the Antarctic Ocean. The prevailing winds on the west of the Andes are south-west and south, which are dry and cool, but on the east the regular easterly trade-winds from the Atlantic are loaded with moisture, and pour down on the slopes of the mountains a copious, and in many places a perpetual rain. In the mountains, at the height of 3,000 to 5,000 feet, the temperature is mild, equable, and healthy, and the inhabitants are noted for longevity. At Huanuco, 5,946 feet above the sea, no chest-affection is known to originate, and the city is resorted to by consumptive patients from other places. Dysentery, and a putrid fever called *tobardillo*, are the commonest maladies, and goître is prevalent, especially among the women. At Lima, 453 feet above the sea, the mean temperature of the year is 73.3°, winter 68.1°, summer 77.6°. The climate is pleasant, and it was formerly reckoned healthy, although the mortality was always excessive from neglect of sanitary measures. Yellow fever has recently appeared, and ague prevails along the entire coast of Peru, at all seasons, and among all classes of the population.

*Bolivia* has three regions of climate,—the *Pano*, high and cold, including the district of Lake Titicaca, 12,846 feet above the sea; the *Paramo*, temperate; and the *Yungas* or valleys, hot. The table-land has clear skies for nine months of the year, and three months of rain. The higher regions are healthy, but fevers prevail in the valleys.

*Chile* has in its central districts a hot dry climate, the thermometer in summer rising to 90° or 95°; on the coast it is finer and more temperate; ice is sometimes seen in winter and spring. Rain falls between June and September south of Copiapo; it is irregular, and often very heavy. Goître is prevalent in the Andes, especially in the province of Mendoza, but otherwise the climate of Chile is noted for salubrity.

The regions of *La Plata*, and the northern and eastern frontiers of *Patagonia*, are characterised by extreme dryness, for the rains carried by the prevailing winds from the Atlantic are exhausted before they reach the interior plains, while those brought by the south-west winds are arrested by the Andes on the west coast of Patagonia, which is excessively moist. Goître is not known to extend further south than about latitude 44°.

### EPIDEMICS.

Diseases of this class are to a great extent limited to the regions of their birth, and in this respect are to be considered as endemic; but they make occasional outbreaks at longer or shorter intervals, and extend over a greater or less extent of surface. In quitting the regions of the torrid zone, or countries having a tropical climate, they lose their intermittent type. The entire class has certain marked peculiarities by which it is distinguished. They always attack a great number of persons at once; they all bear the characteristics of fever; and each resembles the other in having a certain range and a periodical recurrence. It has been ascertained that nearly every epidemic on record was preceded by influenza. Some of these diseases, as yellow fever, are peculiarly fatal to those who are strangers to the soil; while others, as cholera, attack the native and the unacclimatised alike.

1. *Yellow Fever*. The first trace of this formidable malady was observed, at the end of the fifteenth and beginning of the sixteenth century, at San Domingo and Porto Rico; on the continent of South America, and on the Gulf of Darien, at which latter place it prevented the Spaniards from settling. From 1544 there is no record of the disease having broken out anywhere till 1635, when it appeared in Guadeloupe. Thenceforward it occurred at irregular intervals. In the seventeenth century it spread along the continent of South America to latitude 8° south, and in North America to latitude 42° north, but only on the eastern coasts of both. The first notice of it on the Gulf of Mexico was at Biloxi Bay in 1702, and Mobile in 1705. The next was in Pensacola and Mobile in 1765, where it prevailed as an epidemic. In the eighteenth century it appeared on the west coast of South America to latitude 2° south, North America again to latitude 42° north: in Europe, and even in the islands of the Pacific, and in Madagascar. At the beginning of the nineteeth century it extended, in North America, to latitude 47°, in Europe to latitude 48° (the Canary Islands and Leghorn), and penetrated deeper into the American continent than formerly. "Ever since yellow fever attracted attention, or was recognised as a distinct disease from the remittent autumnal fevers of the temperate zone, it has prevailed as an endemic at Havana, raging epidemically from April to December, and occurring sporadically throughout the remainder of the year." From time immemorial it has been indigenous at Vera Cruz, on the Gulf of Mexico. Here its chief victims are strangers who come from colder regions during the hottest season, as well Europeans as those natives who quit the interior for the coast region; and hence it is termed the "strangers' fever". The yellow fever is a disease of hot weather, requiring for its manifestation a high mean temperature. It never appears in any climate where the temperature falls below a certain average. It has often prevailed in almost every town on the Mississippi up to Vicksburg, latitude 32°.24′ north, but has never, except once, reached Memphis, in latitude 35°. It occurs with an early hot summer, when the rain is not copious enough to flood the marshes; but heat alone is not sufficient to engender it.

Among causes hitherto little adverted to, but which by some authors are held to be essential to the production of this fever, is a certain degree of density of the population. Thus Jörg affirms, from personal knowledge and the observation of others, that this fever never occurs in the country or in small villages, but always in large cities, or smaller towns with great trade.

Since this disease is dependent, in a great degree, on an elevated temperature, its occurrence is necessarily limited to the tropical regions, or to countries having a tropical climate. Within these prescribed limits in many places the exciting causes seem to exist, but still the fever has never or seldom shown itself. Its proper seat is the West India Islands and the Bahamas, with portions of the adjoining continents of North and South America. From Brazil to Charleston in one direction, and from Barbadoes to Tampico in another, the exciting causes are in constant though unequal force, depending on different seasons and localities. The fever prevails often, though not generally, in places north of Charleston; visits occasionally the Atlantic cities of the United States, and has ascended as far as Boston; while in the Mississippi Valley it once appeared as high as Memphis. In an eastern direction, but within the same parallels, it has extended to Cadiz, Xeres, Carthagena, Malaga, Alicant, Seville, Barcelona, and other cities on the coast and in the interior of Spain. It has prevailed several times at Gibraltar, once at Rochefort, once at Lisbon, and once at Leghorn. It reaches to between latitude 22° or 23° south of the equator, and to 42° north on the Atlantic coast: to 35° on the western waters of the interior, and to 8°.56 on the Pacific. In longitude it extends from 60° to 97° west, or, including Europe, to longitude 10° east. Until recently, the river Amazon formed its boundary south of the equator; but since 1850 it has invaded Rio Janeiro, Bahia, Pernambuco, and other parts of Brazil. On the Pacific it only appeared once at Panama, twice at Guayaquil, and once at Callao. It does not appear in the East Indies. It has never prevailed in China, Cochin-China, Singapore, Siam, or Ceylon; it prevails only occasionally on the west coast of Africa, Senegal, and the Gold Coast; and has been only three times in eighty years at Cayenne.

From a similar cause, decrease of heat, the yellow fever never appears beyond a certain elevation. At Xalapa, in Mexico, on the same parallel as Vera Cruz, but 4,330 feet above the sea, it is unknown. Maroon Town, and the Phœnix Park, Jamaica, are noted for healthiness, and while the pestilence of yellow fever rages in the low grounds and along the coasts, cutting off thousands annually, these elevated regions enjoy a complete immunity from its effects; for that bane of European life has, according to Major Tulloch, never been known, in any climate, to extend beyond the height of 2,500 feet. The inner Cabrite 430 feet, and the outer Cabrite 590 feet in elevation, are also remarkably healthy. In the island of Grenada, Mount Cardigan, 500 feet, and Richmond Heights, 730 feet, are not sickly. Mount Desmoulin, near Roseau, in the island of Dominica, 1,500 feet above the sea, has invariably been free from yellow fever. The same immunity has been observed in San Domingo, in the mountainous parts of which, whatever be the nature of the soil, this disease does not prevail. In the United States the yellow fever is never known to prevail in very high situations, whatever be the condition of the localities; but at what point it ceases to appear or prevail, is still an unsettled question. The disease varies in intensity, and in the numbers attacked, according to latitude. M. Moreau de Jonnés shows, by elaborate statistics, that in the United States the mortality amounts to one-half of those attacked, while in Spain it is limited to a third or a fourth of the total number. This is accounted for from the difference of climate and soil between Europe and America, which in winter is so extreme, that in order to find in Europe a cold as intense as that of the United States, it would be necessary to remove 12° or 14° farther to the north. The portion of the United States visited by yellow fever has more rivers than Europe, in an equal extent of territory. The more southerly part of it is flat, generally sandy, and covered with the long-leaved pine. Large rivers descend from the Alleghanies; many of them are muddy, and their banks submerged during six months in the year; and the swamps thus formed are covered by thick forests of cypress and other trees. In these marshes rice-fields are established, and it is during the rice harvest in September that the autumnal intermittent fevers appear, by which nine-tenths of the white population are attacked. Of the twelve principal ports of the United States, proceeding from south to north, the first nine, viz., New Orleans, Mobile, Savannah, Charleston, Wilmington, Norfolk, Baltimore, Philadelphia, and New York, unite, in different degrees, the three principal causes of yellow fever; and, consequently, the disease may occur in either or all of them; while in the other three, New Haven, Boston, and Portsmouth, one cause—a great intensity of heat—is wanting, and hence the disease is not often manifested there.

However violent the disease may be at New Orleans, Savannah, or Charleston, no new case ever appears from the day on which it freezes even a single degree. This usually occurs, in the middle States, from the 25th to 30th October, and in the Southern States a month later. It often happens that in the Southern States the disease does not appear at all, if the summer has had frequent alternations of moderate temperature, owing to the occurrence of storms or refreshing rains.

The cessation of yellow fever with the approach of cold weather is so well known, that the farmers of Georgia and and the two Carolinas, who, for security, remain in the country during the hot season, hasten to market with their produce about the beginning of November. They encamp within two or three miles of Charleston, where they wait with the greatest impatience the first appearance of frost, after which, with a feeling of perfect security, they enter the city in a mass, creating the utmost activity in the streets, which, a short time before, were nearly deserted. The more wealthy inhabitants of the Southern States quit the country during autumn to escape intermittent fevers, and the cities to avoid the yellow fever, and spend the sickly or hot season in the Northern States. During the months of December, January, February, March, and April, the lower portions of the Southern States, and their seaports, are extremely healthy.

It is truly astonishing how limited the seat of yellow fever is, and how securely a stranger may live quite in its vicinity if he does not enter the infected circle. La Roche says that the immunity is complete in towns, at short distances from the spot where it is raging. In the pure air of the country it never occurs, however close the intercourse with an infected city; and Drake says, "yellow fever is essentially a disease of towns and cities—an epidemic invasion of the country is unknown." The little town of Guanobacoa, within a few miles of Havana, and with which it is in constant communication, is said never to have had a case of the epidemic; and even in the nearest country-houses around Havana and Casa Blanca, strangers are perfectly safe while the disease is raging in the towns: this exemption is confirmed in the history of the disease at New Orleans, Charleston, Vera Cruz, Matanzas, St. Jago de Cuba, etc. Instances of exemption, even in considerable towns, occur where most unexpected. The town of Cardenas in Cuba, with a population of 3,000, built in a marsh on the north coast of the island, and nearly surrounded by swamps, has never had a single case of yellow fever. Trinidad, in Cuba, has only been subject to the epidemic since its population amounted to 5,000; here, and at Puerto Principe, it appears only at intervals of many years. Matanzas has no marsh, but is situated on nearly dry rock: so long as it was a small place, it had no yellow fever, but now that it has a population of 17,000, has a large trade, and is surrounded by vegetation, it has yellow fever at intervals. New Orleans, although built on piles in a swampy site, has sometimes an interval of exemption during four or five years consecutively; such is also the case with Charleston, and Savannah is seldom visited by the epidemic. In the West Indies the greatest mortality occurs among poor Spaniards from the Canary Islands, Germans and Irish, who have no means of comfort or medical aid. In Havana, sailors are the greatest sufferers, partly from exposure, hard work, and irregular living. Ships which arrive in ballast, and lie long in harbour, are generally the most healthy: coal and sugar laden ships are the least so. In the period of life between 14 and 25 it is calculated that the cases of yellow fever are twice as numerous as at any other age. From 25 to 40 they are still frequent—beyond 40, cases are of rare occurrence; children under 15 are almost exempt: and females are, in proportions, less frequently attacked, and escape more easily than men.

2. *Intermittent Fever, or Ague.* This, the specifically purest form of malarial disease, is an endemic of all warm climates. It has its base within the tropics, and extends northwards till it is arrested by decreasing temperature. Local malaria is the first and most necessary condition of the endemic and epidemic form of the disease, and always exists where the malady prevails, although the peculiar state of the climate and soil may not be clearly ascertained. Intermittents are very prevalent in the West Indies, and in the interior valleys of North America, but are almost unknown in Nova Scotia and the New England States on the Atlantic seaboard. In Europe, ague is endemic on the coast of the Gulf of Bothnia, beyond lat. 62° north. In the interior valley of North America, intermittent fever is the prevailing malady. From its occurring constantly within the tropics, but ceasing far south of the polar circle, it appears that a high temperature is a condition necessary to its production, but this can only be considered as an exciting cause. It is found that a summer temperature of 60° is necessary to the production of the fever, and that it will not prevail as an epidemic where the temperature is below 65°. It therefore occurs in winter at places where the season has a mean temperature of 60° or upwards, as at Vera Cruz, Tampico, Havana, etc.; but at New Orleans, and generally under the thirtieth parallel, where the mean winter temperature is under 50°, the fever is suspended. At New Orleans the necessary heat exists for nine months of the year—March to November ; at St. Louis, five months—May to September ; at Montreal, four, and Quebec, three months. A continuance of more than two months of a heat equal to 60° is necessary to its development ; hence it prevails more in October than April, though their mean temperatures are nearly the same, and its greatest prevalence in every latitude is generally some weeks after the hottest months of the year. It is rarely directly fatal, but frequently results in liver disease and dropsy. The western area of the disease is limited in America on the east by the range of the Appalachian Mountains, into the very gorges of which it ascends, by the valleys which penetrate their flanks : while that of the seaboard extends inland to the eastern base of the same range. South of lat. 33°, where this barrier terminates, its eastern limit is the Atlantic Ocean. On the south-west its boundaries are the Cordilleras of Mexico and the southern Rocky Mountains. It is almost unknown three hundred miles beyond the western boundary of the States of Missouri and Iowa, and above lat. 37° north. On the north it ceases to prevail as an epidemic at lat. 44°, and it does not occur even sporadically at lat. 47°. In Western Europe its limits include Scotland, and on the Continent it extends to the mouth of the Angermann river, lat. 62° 40′, in Sweden. Further eastward it sinks to a lower latitude; and in Central Asia it appears not to extend beyond lat. 55° or 57° north, forming a curve nearly coinciding with the isotherm of 41°. To the south of this, from lat. 54° to 40°, at the level of the sea, on the coasts and river-banks, it constitutes one of the most prevalent diseases. On the shores of the North Sea it causes a mortality of 1 in 20, and even 1 in 14. On the northern boundary it appears only in its more simple form during summer and autumn. Between lat. 55° and 40° it occurs usually in spring as tertian, and in autumn as quartan ague. It is prevalent on the Lido shores and in the Gulf of Venice, but does not enter the city. It is periodical at Rome. Elevation above the level of the sea has a very marked influence on the occurrence of intermittent fever; thus, while it ravages the *tierra caliente* of Mexico, near the level of the sea, it is almost unknown in and around the city of Mexico, 7,450 feet above that level, although both places are in the same latitude. The inhabitants of the Appalachian Mountains, at an elevation of about 3,000 feet, are almost exempt; while those who inhabit the valleys, under the same parallels, are affected. Farther north, at an elevation of 1,500 feet, at the sources of the Alleghany and Genesse rivers, the disease is almost unknown; while on the low shores of Lake Ontario, directly north, it is prevalent. In lat. 41° it is prevalent at 900 feet above the level of the sea. It also prevails at lat. 41°.30 north, at 1,100 feet in elevation, all along the rivers and ponds in the Cuyahoga Basin. The constantly increasing elevation of the desert to the west of the Mississippi, and the increasing dryness of the plains, are probably the chief causes of the disappearance of the fever, under the same parallels in which it prevails on the banks of that river. In Europe, in lat. 52° north, at Cassel, it rises little more than 400 feet above the sea. One degree farther south it occurs every year at an elevation of 600 or 700 feet, near Berka on the Werra; but at 900 feet it comes only once in ten years in isolated cases. In lat. 47°, at Grätz, 1,200 feet above the sea, it is endemic; it is sometimes epidemic at Stanz in Switzerland, 1,700 feet high; and it is prevalent on the plateau of Castile, 2,300 feet high. In Peru, ague is observed at an elevation of 10,000 or 12,000 feet above the sea, and according to Tschudi, it occurs there in dry and barren regions. In Iceland no native is attacked by ague, and strangers suffering from it soon recover: it is unknown in Tasmania.

The geological formation which appears to be favourable for the development of the malady is indicated by Dr. Drake, who says of North America, "the whole southern portion of the cretaceous formation is infested with autumnal fever, beyond perhaps any other portion of the Great Valley"; and again, "like the cretaceous formation, the tertiary region is subject to autumnal fever of a violent character." It has been observed that this fever occurs everywhere in the same geological formation, and in its extension seeks a similar kind of soil. Bierbaum instances the provinces of Alentejo and Algrave in Portugal, where intermittents and remittents prevail, as being similar to South Carolina and some portions of the West Indies. Ague is common among the natives of the Netherlands; it is endemic from the Scheldt to the mouth of the Meuse. At Walcheren, in 1809, two-thirds of the British army were seized with ague, and more than 1,000 died of the disease within the last four weeks of their encampment there.

At Bordeaux, ague is endemic in spring and autumn. Here, when the pools at the west end were drained in 1805, an epidemic ague broke out and seized 12,000 men, of whom 3,000 died in five months.

The influence of sudden atmospheric changes in the production of this disease was exhibited at Landau, where Pauli observed, that on one occasion, when the barometer fell 18° in twenty-four hours, thirty-two individuals were seized with ague within five days.

The decrease of autumnal fevers with the decrease of temperature is strikingly exhibited in the tables furnished at twenty-six military posts between the Gulf of Mexico and Lake Superior. In latitude 24° 33′ north, at Key West, the total number of attacks was—intermittent fevers, 179; remittent, 11; total of the year, 190: while at Fort Brady, latitude 46° 39′ north, the number of cases of intermittent fever was 41, and of remittent 3; total, 44. At Havana it rages epidemically from April to December, and sporadically throughout the rest of the year. The space between latitude 33° and 34° north is traversed by the Cumberland Mountain, a part of the Appalachian chain, forming a rampart more than 1,000 feet in height. This constitutes a climatic limitation to plants, both indigenous and cultivated. In every fertile and well-peopled part of the country south of this rampart, the diseases have a more southern character than on the north, in which direction the tables referred to show a decided and gradual decrease. At Toronto both intermittent and remittent fevers prevail every year, but they are more severe in some years than in others. Simple ague is most common, and intermittents are rare. At London and at Fort Malden, Canada West, autumnal fever still prevails. Intermittent fever is especially a disease of newly-peopled countries, and when it disappears it is because the topographical conditions on which it has depended have been removed. The influence of settlement, cultivation, and town building, is always found beneficial in banishing or mitigating autumnal fever. The prairies, being marshy, are liable to autumnal fever. Open prairie-lands are more healthy than the vicinity of woodlands, probably owing to the greater moisture of the latter. The breaking-up of prairie-land induces fever, but sometimes it does not appear till two and three years after the arrival of the first settlers.

3. *Cholera.* Diseases with choleraic symptoms are observed every year, and at all seasons, in the torrid zone, but most frequently and characteristically in the East Indies, where Asiatic cholera appears to be to the Ganges delta that which the yellow fever is to the delta of the Mississippi, or the plague to the delta of the Nile. Cholera, known from time immemorial, and described in Sanscrit works as an endemic climatic disease, limited to the place of its birth, the delta of the Ganges and the shores of India, was first observed as an epidemic in Bengal, in the month of May 1817; thence it spread first north-west to Mirzapore, next south and south-west, and then continuously in a direction contrary to the monsoons; afterwards it extended in all directions, so that within fifteen months it passed through the whole of India to Bombay. It was influenced in its attacks by the state of the weather, the position of a place, and the means of resistance. In summer it was always more severe than in winter, partly from the greater agglomeration of men in the former season. It was carried by vessels to the great seaports, to the Mauritius, Reunion (Bourbon), etc., where it raged among the ships' crews. At the same time it extended into the interior of the continents, step by step, following the movements of troops and the routes of caravans in all directions. Having gained the great trading cities and fortified towns, it started from them as fresh centres, till it overspread all Asia from Aleppo and Bagdad to Pekin. Thence it reached Ceylon, the Philippines, Moluccas, and Sunda Islands. In the East its victims were countless. At Muscat in Arabia, and its vicinity, for example, more than 125,000 persons perished.

The great movements of troops occasioned by the wars of the British with the Indian tribes,—of the latter with Persia, of Persia with Russia, and of Russia with Poland, brought the disease systematically into Europe; and it is calculated that in the Russo-Turkish campaign, cholera was, to both armies, ten times more destructive than the bayonet or the bullet. At the end of that war it was expected that the disease would be arrested by the removal of the troops from infected districts, and by the approach of winter, but it was found necessary to convey some Russian troops from Persia and Turkey to Poland, and thus cholera reached the banks of the Vistula. The Austrian and Prussian quarantine systems were useless, and so it spread from St. Petersburg to Odessa, by ships throughout Europe, the whole of which was visited by the epidemic, except such countries as Saxony, some of the regions of middle Germany, and others inclosed by mountains, or where little commercial intercourse is carried on. From Europe cholera speedily crossed the Atlantic with the great tide of emigration towards the United States; here it showed itself in its most virulent form, especially in the ports where emigrants landed, but at first it did not extend much into the interior. The first ships with cholera patients on board arrived at New York and Quebec in 1832. At New York a kind of quarantine was kept up, and many died in the hospitals. In Canada, as in Europe, none was attempted, and it spread through the larger towns, and along the commercial routes to the States of the Union. Chicago, on the south coast of Lake Michigan, the chief port for Canadian emigrants, was attacked first, and most severely. The next point whence it spread through the States was New Orleans, to which it was carried by ships from New York, shortly after its arrival there. At New Orleans from 80,000 to 100,000 emigrants arrive annually from Europe, and there is no quarantine. Food for the disease was supplied by the arrival almost daily of hundreds of new emigrants, of whom often thirty died in one vessel on the voyage from England, Germany, or France. It ravaged Lafayetteville, a suburb of New Orleans inhabited chiefly by Germans and Irish, and was carried quickly by steamers to Louisville and Cincinnati on the Ohio, both, but especially the latter, chief landing-places for emigrants. Here the mortality was frightful, greatly owing to a kind of fatalism, which led the Germans and Irish to believe that it was useless to attempt precaution or remedy, farther than by wearing amulets, and confining their diet to herrings and cold water. From Cincinnati the disease extended up the

Ohio to Pittsburg, and spread thence to New York, Philadelphia, and Baltimore, where, fortunately, it was arrested by the sea. At the same time it was carried by steamers to Albany on Hudson Bay, where it was very severe. From New York it spread little at first; and, while it extended from Europe to New Orleans, and thence to St. Louis, Louisville, Cincinnati, and New York, numerous cities which lie between, on the Mississippi and the Ohio, remained either altogether free, or were only overtaken long after the outbreak of the disease in places more than a thousand miles to the north and east of them. In the vicinities of great cities it often did not appear till after it had travelled to places many hundred miles distant. It passed from New Orleans to Texas before it showed itself in the vicinity of the city, and from St. Louis it was conveyed by travellers to California, among the Indians, to Independence and Fort Leavenworth on the Missouri, and thence west to Fort Laramie, near the Rocky Mountains, and all this in less time than it took to reach Belleville, within a short distance of St. Louis. The whole of the southern States of the Union, with the exception of some of the cities on the Ohio, the Cumberland and Red Rivers, or large ports, as Baltimore, almost entirely escaped the cholera. At the ports of Galveston and Lavaca in Texas, where troops arrived from New Orleans, it first appeared in 1848; hence it accompanied the gold-diggers into the interior, to Austin. Shortly after its appearance in Galveston, it was conveyed by ships from New Orleans to Corpus Christi and Brazos St. Jago, at the mouth of the Rio Grande, and accompanied the caravans and steamers along that river.

Matamoras was next visited, in summer; and thence, extending westward, the cholera attained the height of 5000 feet above the sea at Chihuahua, and a still greater elevation in the district of Paso del Norte on the Rio Grande. In the former city, from ignorance of its treatment, it carried off almost every one who was seized, amounting to 60, 70, and even 107 in one day. The States of Ohio, Illinois, and Missouri, suffered most of the whole Union; not only because the chief emigrant ports are situated in them, but because they are traversed by the principal navigable rivers. The most fatal places were in Ohio, the district between Sandusky and Cincinnati; in Illinois, the vicinity of Belleville, the towns on the Mississippi, on the Illinois, the canals, and Lake Michigan; and in Missouri, St. Louis and its vicinity, the principal towns on the Missouri, and the routes leading to California. The frequent appearance of cholera along the courses of rivers led at first to the hypothesis, that the disease had a peculiar affinity for water; but the fact was overlooked, that great cities, necessarily the centre of epidemic virulence, are mostly situated on navigable streams; and the triumphant march of the cholera-plague along the caravan routes in Asia and America, where no water was met with for many days in succession, evinced the fallacy of the opinion. This great epidemic spanned the entire globe: unlike the yellow fever, it was not confined to a certain elevation above the earth's surface, but, leaving the lower stratum of the atmosphere, it followed its victims to the summits of great elevations, as in the table-land of Malwa, the villages of which are situated 2500 feet above the level of the sea. In 1818 it appeared at Catmandoo in Nepaul, at the foot of the Himalayas, nearly 5000 feet above the sea; and in 1822, and again in 1852, it raged with great violence at Erzeroum, 6100 feet above the sea. Yet it appears that difference of level has a certain influence on the frequency and virulence of the disease. In the higher quarters of Paris, with an elevation of 56 feet above the river, the deaths were at the rate of 18·55 per 1000, while in the lower quarters, 9 or 10 feet above the Seine, the deaths were 23·60 per 1000. In London, the deaths were nearly in the inverse ratio of the elevation of the ground: under 20 feet above the Thames the proportion was 102 in 10,000; from 20 to 40 feet, 65 in 10,000; from 40 to 60 feet, 34 in 10,000; and from 60 to 80 feet the mortality was 27 in 10,000; 80 to 100 feet, 22 in 10,000; at 350 feet, only 8 in 10,000. In a single year England lost, by the visitation of cholera, 70,000 individuals, of whom 30,000 were adults, being 10,000 more men than fell in battle in the wars between 1800 and 1815.

4. The *Plague* has its endemic seat on the eastern shores of the Mediterranean, where it has been known to exist since the middle of the sixth century. Pariset considers the Nile delta as the peculiar developing place of the plague. The causes to which it is attributed are, the great heat, the overflowing of the river, the want of cleanliness in the people, and the exposure of the dead in and near their dwellings. It may be considered as occupying permanently a portion of the Old World, extending between the parallels of latitude 29° and 42° north; and while it is thus permanent in some places, it appears more or less frequently in others. Its term of periodicity was reckoned to be, for Constantinople 9 years, Egypt 5, Aleppo 10, Antioch 15, and Cadiz 43 years. In Sydenham's time it was said to ravage England every

40 years. It has not appeared in Scotland since the reign of Charles II, although it remained a few years longer in England. It seldom extends to the southward beyond Siout in the valley of the Nile, or Jiddah on the Red Sea. In Asia it prevails chiefly on the coasts of Syria, and a portion of the shores of Asia Minor, where it sometimes ascends the river valleys. In Europe it is endemic only on a part of the eastern coast of Turkey. In 1816 it was very destructive in the Ottoman empire, and extended into Austria, Italy, and Sardinia, and it was at Moscow and Marseilles last century. In 1841 it raged in Syria and at Erzeroum with great violence. It has never yet appeared in the southern hemisphere, nor in America.

Like the yellow fever, the plague appears to be limited to the lower portions of the earth's surface, the more elevated situations being usually exempt from its scourge. When it is ravaging the lower quarters of Constantinople, the inhabitants of the higher portions of the seven hills on which the city is built often escape altogether; and Brayer mentions a village situated on mount Alem Dagh, at an elevation of about 1600 feet above the sea, where it was never known to appear, and which was resorted to as a place of refuge for the citizens; and there is a place in Malta hitherto inaccessible to the disease, and on this account called *Safi* (pure). It is recorded by the French physicians, that during their occupation of Cairo, the plague never reached the citadel of that city; and Clot Bey states that it, as well as the village of Loumeldik, situated at a considerable elevation, was spared during the epidemic of 1835. The nature of the soil has much to do with the development of this disease. As an argillaceous soil is most favourable for the development of malarial fevers, so it is a characteristic of the localities where the plague is endemic. Pugnet says that it rarely appears in deserts or sandy soils, but that it immediately breaks out on the rupture of the dyke which confines Lake Madieh; and Clot Bey observes that, during the great epidemic plague of 1835, the Egyptian regiments encamped in the desert escaped almost entirely, notwithstanding the maintenance of communication with the capital and other places where it committed the greatest ravages. Ghizeh, which is placed on the banks of the Nile, and completely inundated by the river, is much more frequently infected than Cairo. Salahieh does not receive its contribution of Nile water till long after the capital, and it is not visited by the plague till after a similar interval. According to Gaetani Bey, the plague is arrested at Assouan, on the borders of Nubia, on account of the difference of situation, heat, dryness, and the nature of the soil. It breaks out readily in localities where the water is stagnant from the absence or neglect of canals; hence Bassorah and Bagdad, formerly safe through the cautious administration of the canals, are now the theatre of this scourge.

When the plague visits Constantinople, it appears usually between the 1st and 20th of July, and decreases on the approach of winter; while in Egypt it commences in winter, and disappears at the end of June.

5. *Typhus*. This form of fever, which occurs frequently as an epidemic, appears to belong exclusively to the north temperate zone, and even here it avoids extreme latitudes. It is scarcely ever mentioned by medical voyagers in hot countries. As yellow and intermittent fevers occur in low latitudes, near the level of the sea, so typhoid fevers have their base line in a high latitude, and at a greater elevation. Yellow and intermittent fevers decrease from south to north, but typhus, on the contrary, decreases from north to south. In America, typhoid fevers diminish in frequency beyond the parallel of 45° north. Typhus does not appear among the fur stations of the Hudson Bay Company between the parallels of 48° and 58° north; and no mention is made of its occurrence among the crews of the Arctic voyagers nor among the Esquimaux, who live in close unventilated snow huts; neither has it been observed by Ermann and Wrangell among the inhabitants of Siberia. Typhus has, therefore, a northern as well as a southern limit. In Western Europe it prevails between the parallels of 44° and 60° north, or between the isothermal curves of 48° and 52°; and in North America between the parallels of 32° and 48°. In places where the mean annual temperature rises above 62°, or falls below 40°, it prevails but little in either continent. The geographical and climatal limits of typhus in Europe and America will be found to correspond nearly with those of the glutinous cerealia and the potato. It decreases with elevation; and to this cause has been attributed its absence in the hospital of Madrid, 1995 feet above the sea. It occurs in every season, but is most prevalent in autumn and winter. According to the army reports, typhus appears to be three times more prevalent in Lower than in Upper Canada. As intermittent fever is a disease of new, so typhus is of old countries; where the soil has been longest cultivated, typhoid fevers are most prevalent. The early settlement of the lands along the estuary of the St. Lawrence is probably the chief cause of the remarkable prevalence of typhus, and the absence of intermittent fevers there.

During 1847, the so-called famine-typhus prevailed over a great portion of Europe. In Poland, especially in Galicia, and in Silesia, it was currently reported that 40,000 of the people were in a state of starvation. In Ireland it carried off 100,000 of the population. It commenced in Cork and Liverpool, where thousands of emigrants were assembled on their way to America. The wellbeing of the great trading towns prevented the spread of the disease on shore; but the good effects of sufficient food on ship-board were counteracted by the foul air of the crowded space between decks, and a fatal and highly contagious form of typhus was engendered among the passengers, which, in spite of quarantine regulations, they conveyed into the principal ports of Canada and the United States, whence it spread to the interior of the country. In Quebec, Montreal, New York, and New Orleans, not only the emigrants, but also the inhabitants, fell in thousands before the scourge. In New Orleans the "ship's fever," as it was called, was more dreaded than the yellow fever. The approach of winter, and a strict enforcement of quarantine laws, put a period to the epidemic in 1848.

6. *Phthisis.* Tubercular consumption cannot be said to be a disease peculiar to any one portion of the globe, or to be dependent on climate in any appreciable degree, unless it can be shown that it does not prevail in the excessive climates of the north. It originates in all latitudes from the equator, where the mean temperature is 80°, with slight variations, to the higher portion of the temperate zone, where the mean temperature is 40°, with sudden and violent changes. The opinion long entertained, that it is peculiar to cold and humid climates, is founded in error. Far from this being the case, the tables of mortality of the army and navy of this and other countries, as well as those of the civil population, warrant the conclusion that consumption is more prevalent in tropical than in temperate countries. Consumption is rare in the Arctic regions, in Siberia, Iceland, the Faroe islands, the Orkneys, Shetlands, and Hebrides. And in confirmation of the opinion that it decreases with the decrease of temperature, Fuchs shows, from extensive data, that in northern Europe it is most prevalent at the level of the sea, and that it decreases with increase of elevation to a certain point. At Marseilles, on the seaboard, the mortality from this cause is 25 per cent.; at Oldenburg, 80 feet above the sea, it is 30 per cent.; at Hamburg, 48 feet above the sea, it is 23 per cent.; while at Eschwege, 496 feet above the sea, it is only 12; and at Brotterode, 1800 feet above the sea, 0·9 per cent. It is calculated that in the temperate zone, within which nearly all the civilised inhabitants of the globe are located, at least one-tenth of the population die of this malady. It is uniformly more fatal in cities than in the country: in England the excess in cities is equal to 25 per cent. The greatest mortality occurs from the age of 15 to 30: taking the sexes together, it destroys one-half of all who die from every kind of disease in Massachusetts, between these ages.

7. *Cretinism.* Wherever cretinism occurs, goître is generally present; but in many places where goître prevails, not one cretin is to be found. Although only recently brought into notice, cretinism was known and described early in the seventeenth century. Though sometimes sporadic, it is chiefly an endemic disease, supposed to originate in certain conditions of soil, air, and water, and occurring in deep narrow valleys of Alpine regions, as well as in low, flat, marshy districts: in the former it is termed *Cretinismus alpinus*, and in the latter, *Cretinismus campestris*. This dreadful malady, which has been termed the "Leprosy of the age," is thus defined by the Sardinian commissioners:—"Cretinism is a degeneration of the human species, which manifests itself in certain parts of the globe, and is characterised by a greater or less degree of idiocy, associated with a vitiated state of the body." They divide the sufferers into the complete cretins, simply vegetating masses, devoid even of instinct; the half cretins, who can articulate some words, can make themselves understood by gesticulations, and can perform some mechanical labour; and the cretinous, who are possessed of understanding and will. Cretinism differs from simple idiocy in that the body of an idiot is often well formed, while the cretin is an idiot whose physical formation has suffered a general degradation. In the complete idiot the faculties are radically extinct, or exist only in a rudimentary state, whilst in the cretin they are oppressed and overpowered by the disease, but not abolished. The cause of this disease has been sought for in all the natural elements, by turns, without any satisfactory result. In the province of Kumaon in India, goître is so prevalent that one-half of the population is afflicted by it. A large proportion of these cases occur among the population who live on the limestone formation; whilst goître is almost unknown on the granite, gneiss, and sandstone formations. One in every three of the population on the limestone formation was afflicted with goître, and one in twelve was a cretin. On the mica, hornblende, and sandstone formations, neither goître nor cretinism was seen. Hence the disease is produced, according to some authors, by the use of water impregnated with lime. Others refer it to exhalations from the soil, electricity, drought, etc. Again, a specific malaria is contended for as the only way to account for the appearance of the malady in one part of a valley, a village, or even a single house, while in the immediate vicinity it has never appeared. In Europe, cretinism occurs in the valleys of the Pyrenees, the Alps, Apennines, the Schwarzwald, the Rauhe Alp, Thuringer Wald, the Harz, the Erz, and the Riesen-gebirge, in the Carpathians (Neusohl in Hungary), among the mountains of Styria, the Vosges Mountains in France, in the West of England, in Wales, in Denmark, and the lowlands of Rhenish Prussia: and it has recently been observed in Berlin, Paris, Vienna, and other populous places. It prevails in the mountainous countries of Upper Asia, in the Cordilleras of South America, and in the northern part of North America.

Saussure was the first to remark that cretinism is limited to a certain height above the sea. This he fixed at 1,000 metres (3,280 feet) in Switzerland; and numerous observations have since confirmed the general accuracy of his statement, which is exemplified in the total absence of cretins at Ursern, in the Canton Uri, 4,600 feet above the sea, where, in 1847, not one was found in a population of 4,000. In ascending the St. Bernard from Martigny, the disease decreases in proportion as the surface rises, till, at the highest village, St. Pierre, no trace of it is to be found. The Sardinian Commission dispute this statement, and account for the number of cases occurring under 3,200 feet, by the circumstances that the cultivated soil and the habitations of men are found under this level; and they quote many villages in Savoy much higher than this, which are peopled with cretins. But all observation shows that this limit changes as in the case of other diseases, according to geographical position. Thus in Southern Germany it varies from 1,400 to 2,100 feet; in Würtemberg it is about 2,100 feet; in Sardinia, 5,300 to 6,400 feet; and in the Cordilleras of the Andes, according to Humboldt and Boussingault, 15,000 feet. Exceptional cases occur in Switzerland above the height named, and the Sardinian Commissioners themselves state that the most elevated parts of Upper Savoy are entirely exempt from goître and cretinism. It is calculated that in Switzerland there are in all 20,000 cretins, and these are very unequally distributed; for while in the Canton Valais there is one cretin in every 25 of the inhabitants, in that of Uri there is 1 in every 83, and in the Canton Glarus only 1 in 375. In 1845 the population of Sardinia was 4,125,740, half of whom occupy the mountainous districts where cretinism is endemic. The number of cretins is stated by the commission at 7,084, of whom 5,500 were in the provinces of Savoy and Aosta, 1,413 in Maurienne, and 2,180 in the valley of Aosta.

From the inquiry instituted by the king of Würtemberg, conducted by Dr. Rösch, it appears that in a population of 1,726,536 souls, 5000 families were more or less affected by the disease, of whom 1000 were cretinous-deaf, and 144 cretins of the highest degree, destitute even of the appearance of humanity. Baden had, in 1844, 440 cretins, of whom 275 were complete, and 165 half cretins; in 1847 the total number had increased to 490. The official returns of the population of France for 1851 show that 42,382 of the inhabitants were goîtrous (not distinguishing cretins), equal to 118 in 100,000 individuals; these were mostly in the departments Hautes Pyrenées, Hautes Alpes, Ariége, Vosges, and Puy de Dôme.

The government of Bavaria instituted an inquiry in 1840; and Professor Virchow reports, that in Lower Franconia alone, in 500,000 of a population, there are at least 200 true cretins. It appears that the disease is most prevalent in the highlands of Bavaria, but statistics are not yet published on the subject. In Upper Austria the disease is so prevalent along the banks of the Danube, that, according to Dr. Schauzberger, whole families consist entirely of cretins and half-cretins; so that, in villages with a population of 4000 to 5000 souls, not one man is found capable of bearing arms. And the statistical inquiries instituted by the Archduke John show, that in Styria there are 6000 cretins of the highest grade. In Rhenish Prussia, cretinism is endemic in the lower districts. Of this the small island of Niederwörth, below Coblenz, furnishes a remarkable example. Of 750 inhabitants, nearly all are goîtrous, and 40 are cretins. The other places where it is most prevalent are the vicinity of the Lacher-see, near Bonn, and the village of Niedermendig, celebrated for its millstones, where there are 22 cretins in a population of 300. In Denmark, Dr. Huberts states, that among 2000 idiots there are many true cretins. These are found mostly on the north sides of the valleys, as in the Canton Argovia, in Switzerland. In England, cretinism is endemic in Somersetshire, where the village of Chisleborough, situated in a valley, has 350 inhabitants, the greater part of whom are goîtrous, and 24 are cretins. A village in Yorkshire, with 200 inhabitants, has 20 cretins. The Netherlands are remarkably exempt from this form of disease.

8. *Goître* occurs in all quarters of the globe. In Europe it is prevalent in the valleys of the Pyrenees, on the Spanish more frequently than on the French side, where the mountains are more steep; but the plains of Spain, as well as the highest inhabited parts, are free from the disease. It is endemic in all the mountainous countries of middle Europe, from the French coast to the shores of the Black Sea. It also appears sporadically in the plains of middle Europe, at Berlin, St. Petersburg, and in other parts of Russia; but the marshy districts of Russia on the North Sea are exempt from goître. In England it is common in Warwickshire, Lancashire, Somerset, and Derby; in the latter county it is so prevalent that it is termed the "Derby neck." It is said to occur in Scotland, in the island of Arran and in Peeblesshire.

Its northern limit is in Western Norrland, Sweden, about latitude 63° north. In Asia it occurs in the mountain valleys of the Ural, the Caucasus, the mountains of Lake Baikal in Central Asia, in the Himalayas, and in Sumatra. In Africa goître has been observed in the Atlas Mountains, and in the mountains of Kong. In North America it is very prevalent: its northern limits appear to be about latitude 58° north, at the sources of the Peace River. It is common on the banks of the Saskatchewan River, in Upper and Lower Canada, in the States of New York and Pennsylvania, and especially on the tributaries of the Ohio, where the Indian women are generally goîtrous. In South America it extends to latitude 44° south, in the valleys of the Cordilleras, and also in Brazil. In its prevalence above the level of the sea it closely resembles the distribution of cretinism.

9. *Leprosy.* In its endemic distribution, this malady extends over South America to latitude 30°, excluding, therefore, the southern portion. In North America it occurs in Greenland; and in Europe it is limited to portions of Greece, Norway, Iceland, and Lapland. In Africa it spreads to latitude 20° south; it was frequently observed in the upper Niger; and it prevails over the western and south-eastern parts of Asia, with the adjacent islands. Its greatest intensity corresponds with the belt of maximum heat of the globe.

According to Pliny, *lepra nodosa* appeared in Italy and other parts of Europe in the second half of the second century B.C. In consequence of the warlike expeditions of the Saracens and the Crusaders, it became so general, that at the end of the thirteenth century, 19,000 hospitals for lepers were opened in Middle and Western Europe. Here it remained till the fifteenth century, when it invaded Russia, and extended to Iceland and Greenland. Leprosy had, therefore, left its original endemic centre, and spread in a direction from south to north. Afterwards it gradually declined, and is now of comparatively rare occurrence in Europe and North America. The malady is however common at Bahia in Brazil. It is probable that the disease in the Western Hemisphere is the same as that of the East, or a similar malady modified by climate.

10. *Pellagra, Italian Leprosy, or the Lombardo-Venetian Plague,* is a disease which has baffled all attempts to discover its origin. Its usual course is lunacy, mania, or helpless imbecility, and death. In 1831 official returns showed that 20,000 Milanese—amounting to one-third or one-fourth of all the patients in the Lombardo-Venetian lunatic asylums—were attacked by pellagra; and in 1843 the proportion in the hospitals of Brescia had increased to three-fourths of the patients. This loathsome malady is described as being much more deplorable even than cretinism: it sometimes keeps the patient in a hopeless state for ten or fifteen years. It has been ascribed to the use of maize, which forms the principal article of diet to nine-tenths of the Milanese peasantry; but it does not occur in the similar maize-growing districts of Naples, Sardinia, and Sicily, nor even in some districts of Lombardy itself. It appears to be a local disease, and is cured by removal and nourishing animal diet. Some years since the pellagra appeared in France, in the department Gironde, and in Gascony. Its limits of greatest intensity are between latitude 43° and 46° in Upper Italy, the south of France, and the north of Spain.

### THE ARMY.

Since a great part of our information regarding diseases incidental to tropical countries is derived from tables of the sickness and mortality of European soldiers and sailors, it becomes important to inquire how far the morbid phenomena therein exhibited may be due to other causes than the direct effects of climate. It has been usual to attribute a large proportion of the mortality among troops to the use or abuse of intoxicating beverages. Thus Forey says, in reference to the troops in America—" The vice of intemperance is the most prolific source of disease and death;" and again, " nine-tenths of the mortality at the salubrious posts along the coast of New England has its origin in inebriating potations." Similar statements constantly occur in the returns of the British army; and hence it is inferred that all stimulating beverages are hurtful in warm climates; but these statements, so injurions to the reputation of the soldier, are, it is believed, greatly exaggerated.

The deficiency of nutritious food is a frequent cause of mortality among troops. The use of invigorating nourishment is a primary condition of successful defence against malarial poison. Next to food, and perhaps before it, in importance in the development of disease, is the water, often stagnant or corrupted by exposure to the sun, or containing a large amount of salts, with which the soldier is obliged to satisfy his thirst. Besides physical causes, the soldier is subjected to moral influences, such as expatriation, chagrin, and many others, single or combined, which originate or increase derangement of the functions. To these may be added the unhealthy situation, overcrowding, and bad arrangements of the barrack accommodation in tropical countries, and the fatigue and exposure incident to the events of war, of which the returns of the British, French, American, and Prussian armies afford many instances. During the first occupation of Oran, in Algeria, the French army lost 1 soldier in 12, and 1 officer in 54, and this rate continued for several years; but in 1837 harassing expeditions commenced, and the rate of mortality from disease increased to 1 private in 9, and 1 officer in 42. When all was again tranquil, the mortality was reduced to 1 private in 19, and 1 officer in 90.

These circumstances, in great part peculiar to the army, render a comparison between the diseases incident to them and civilians in the same climates impracticable; and even a comparison of the liability to disease among soldiers of different armies is extremely difficult, since not only the difference of age at entry, and the longer or shorter period of service, but also the nationality of the soldiers, must be taken into the account.

On the other hand, many circumstances in an army favour this comparison, as the exclusion of the weak and sickly from all the returns, the same occupations, and a similarity in lodging, dress, and food—circumstances which no civil population can present on so large a scale. In the British possessions, France, and America, it is found that the mortality among troops, even when in their own country, greatly exceeds that of the civil population; and that in the infantry the mortality is much greater than in the cavalry and artillery. According to Casper, the soldier and civilian in Prussia stand in precisely the same relation as regards mortality. He states from official statistics, that the mortality in the Prussian army averages only 13 per 1000. In Great Britain, the ratio is upwards of 15 per 1000; and Count Morozzo shows that among the infantry of the Sardinian army it amounts to 35 per 1000.

In the Prussian army, while the infantry, as stated, die at the rate of 13 per 1000, among the artillery the ratio is 10, cavalry 9, and the pioneers only 6 per 1000. This proportion holds in the British and French armies. Among the Sardinian troops it is still more striking; for, while in the infantry it amounts to 35 per 1000, in the cavalry it is only 18 per 1000. This appears to indicate that the infantry fall faster, because they have to bear the burden of the service alone, while the cavalry and artillery share it with their horses. The influence of climate on the health of troops is shown in the following table. The effects of discipline, or of undetected moral causes, are strikingly exhibited in the comparative number of suicides among the different corps. In Britain, of 10,000 men, 8 die annually by their own hands; in Prussia, only 4 in 10,000. In Britain, the greatest number of suicides occur among the cavalry; and this is also the case in Prussia, where among the artillery and pioneers the proportion is 2, the infantry 4, and the cavalry 7 in 10,000.

*Comparative Statements of the Mortality among the British Troops serving in different parts of the Empire.*

AVERAGE MORTALITY PER THOUSAND OF WHITE TROOPS ANNUALLY.

| COLONIES. | For 20 years ending in 1836. | For 10 years ending in 1846. |
|---|---|---|
| New South Wales | 14 | 11 |
| Windward and Leeward Islands | 78·5 | 68·7 |
| Jamaica | 121·3 | 66·9 |
| Gibraltar | 21·4 | 10·9 |
| Malta | 16·3 | 14·9 |
| Ionian Islands | 25·2 | 15·5 |
| Bermudas | 28·8 | 29·2 |
| Nova Scotia and New Brunswick | 14·7 | 13 |
| Canada | 16·1 | 12·6 |
| Newfoundland | 14 | 9·1 |
| St. Helena | 34·2 | 15·4 |
| Cape of Good Hope | 13·7 | 13 |
| Mauritius | 27·4 | 24·4 |
| Ceylon | 69.8 | 41·4 |

This Table shows a great saving of life during the last ten years.

In the year 1849, the ratio of mortality among the white troops, in our different colonies, was as follows, showing, in many instances, a great discrepancy with the ten years average above:—

In Australia, 8; British Guiana, 14·2; Trinidad, 33; Tobago, 98·6; Grenada, 12·3; St. Vincent's, 6; Barbadoes, 128·8; St. Lucia, 17·4; Dominica, 40·4; Antigue, 10·9; St. Kitt's, 19·4; Windward and Leeward combined, 68·4; Jamaica, 48·3; Gibraltar, 8·4; Malta, 30·1; Ionian Islands, 23·1; Bermuda, 8·4; Newfoundland, 10·3; Nova Scotia and New Brunswick, 19·7; Canada, 15·6; St. Helena, 8·4; Cape of Good Hope, 13·3; the Mauritius, 14·6; Ceylon, 21·5; Madras, 22·4; Bengal, 61·3; Bombay, 26·6.

Comparing the foregoing mortality with that of the troops in the United Kingdom, the superiority of the Australian climate will be manifest.

AVERAGE MORTALITY PER THOUSAND OF TROOPS EMPLOYED.

| UNITED KINGDOM. | For 7 years previous to 1836. | For 10 years ending in 1846 |
|---|---|---|
| Household Cavalry . . . . | 14·5 | 11·1 |
| Dragoon Guards and Dragoons . | 14·3 | 13·7 |
| Foot Guards . . . . . . | 21·6 | 20·4 |
| Regiments of the Line . . . | 18·5 | 17·9 |

Among the French troops in Algeria, from 1837 to 1846, the loss was, in proportion to those serving in France, as 77·8 to 19·5, or nearly as 4 to 1. Since the illusion of the supposed power of acclimatisation to abate disease in hot climates has been detected, and the system of rotation, limiting the residence of a body of troops to three years in the same colonial region, has been adopted, a great decrease of mortality has resulted at all the stations. In Gibraltar this decrease was from 22 per 1000 before, to 12 per 1000 after the change of system. For several years the mortality among medical men on the west coast of Africa was 78 per cent. annually, and no one could be got to supply the vacancies; but when the time of residence was limited to one year, the mortality immediately fell to 25 per cent.

It is a common prejudice, even in the army, that the number killed in battle exceeds the mortality from disease; but this is so far from being the case, that the loss of the French army in Egypt, at the beginning of the present century, was, killed in battle, 3614; mortally wounded, 854; killed by various accidents, 290; died by disease, 4157; total, 8915.

The British army in Spain lost in 41 months—January 1811 to May 1814—on a force of 61,511 men, 24,930 by disease, and only 8,889 by the fire of the enemy. The same rule holds good in the navy.

### THE NAVY.

The *Home station* of the British navy embraces the flag-ships, revenue-cutters, etc., on the shores and in the harbours of Great Britain and Ireland. The *Mediterranean command* comprises the whole of the Mediterranean Sea, from the Strait of Gibraltar to the Gulf of Scanderoon, and the shores of Spain and Portugal north to Lisbon. The *Cape of Good Hope command* includes the east and west coasts of Africa; and the *East India command* extends from the Isthmus of Suez to Tasmania; but the operations of the squadron are principally directed to the Bay of Bengal, the coast of Coromandel, and the island of Ceylon. The *North American* and *West Indian command* extends from the southermost shores of the Spanish Main to Labrador, including the Windward and Leeward Islands and the whole coast of the Spanish Main. The *South American command* comprises the east and west coast of South America, and the western shores of North America, with an occasional extension to the Sandwich, Marquesas, Society, and Friendly Islands.

The diseases incident to sailors are the same as those which prevail in the countries off which they are cruising. Thus, on the North American and West Indian station, and in the West African command, the rate of mortality from fevers and dysentery is comparatively high; while off the south-east coast of Africa, the whole of South America south of the equator, and the northern shores of North America, which are all very healthy, it is remarkably low. The comparative amount of sickness and mortality from consumption is shown in the following table, which gives the average for seven years, from 1829 to 1836:—

| STATION. | Number attacked per 1000 of force | Died per 1000 of force. |
|---|---|---|
| East Indies . . . . . | 2·9 | 1·2 |
| Home . . . . . . . | 4·1 | 1·4 |
| South America . . . . | 3·2 | 1·5 |
| Africa . . . . . . . | 3·4 | 1·5 |
| North America and West Indies . | 4·8 | 1·9 |
| Mediterranean . . . . | 5·1 | 1·9 |

From this it appears that the mortality from this disease was least in the East Indian, and greatest in the West Indian and North American force; but the loss in the greatest, being under 2 per 1000 annually of those employed, is not heavy when compared with that of other portions of society at corresponding ages. Although the proportion of mortality in the West Indian and North American and the Mediterranean commands was the same, the proportion of attacks in the latter preponderated. From the returns it appears, that the Mediterranean station of cruising-ground is the least favourable of any to consumptive disease; for, besides the greater frequency, there was also a larger proportion of invaliding. The East India station was most favourable for diseases of the lungs, but least so for dysentery. On the Home station the deaths from this cause amounted to only 3 in 56 attacks; in the East Indies the ratio 4·2 per 1000 annually; in the African, 3·4; in the South American, 1 per 1000 annually of force; and in the West Indian and North American commands, and the Mediterranean, it was 1 in 3500 each.

Nothing can be more striking or satisfactory than the results of sanitary measures in the British navy. Formerly scurvy, putrid fevers, and ulcers, were considered inseparable from a life at sea. In 1741, the *Centurion* ship of war lost 200 men out of 400 from scurvy, on the South American station, which is now as healthy as the Home station. In 1797 the victualling of the navy was changed. Abundance of wholesome food and good water was supplied, and immediately the health of seamen strikingly improved, and this improvement has been regularly progressive. In 1779, one out of every eight seamen employed in the navy died; in 1811, one out of 32; in 1836, one out of every 72. Or in other words, 76 years ago the mortality was at the rate of 125 per 1000 annually of the force employed; 50 years ago, 31; 15 years ago, 13 per 1000; and now (1856) the lifetime of sailors is not only far beyond that of soldiers, but the chances of longevity, in a well regulated life at sea, are at least equal to those in the most favoured regions ashore. Between the years 1780 and 1783 the comparative mortality in the British navy was, from disease, 3200 men; died in battle, 640; died by wounds, 500. During the three years of active hostility on the coasts of China, 1840-1843, only 29 men fell by the hand of the enemy, while 748 perished from other causes, chiefly diseases produced by climate.

[From anxiety to print the whole of Mr. Johnston's learned paper in one fasciculus of the *Transactions* of the Epidemiological Society, the Annual Report of Council, and the Report of the Epizootic Committee on Pleuropneumonia, are necessarily postponed. Mr. Johnston's paper has been written for publication in his valuable *Physical Atlas*, where it is accompanied by a map, in which the facts laid down are carefully delineated. It is no false praise to say, that no scholar out of the domain of medicine has ever before contributed so valuable a document to medical literature. The members of the Epidemiological Society feel that Mr. Johnston's contribution to their *Transactions* of a paper so rich in research, and intended exclusively for his own masterly work, is an act as graceful as it is generous.]

